# Between illness and health: A scoping review of cancer experience through the construct of liminality

**DOI:** 10.1177/13591053251351807

**Published:** 2025-07-28

**Authors:** Thierry Mathieu, Nicolas Favez, Sarah Cairo Notari

**Affiliations:** University of Geneva, Switzerland

**Keywords:** cancer, in-between state, liminality, psycho-oncology, scoping-review

## Abstract

Individuals affected by cancer (IAC) often oscillate between two states during or after treatment: feeling ill/cured or dying/living. This in-between state relates to liminality; however, the construct is used differently from one study to another, which creates indeterminacy. Our scoping review aims to clarify how liminality is theoretically defined and applied in psycho-oncology to describe IAC’s experiences and identify the associated concepts. We searched five databases using English and French keywords, selecting 20 peer-reviewed studies from 454 retrieved. Studies associated liminality with words such as state, space, or experience. They used often liminality in contexts involving psychological, social, or physical difficulties. Despite differences across studies, convergent points emerged. We propose a tentative definition of liminality: a state where IAC face significant difficulties following cancer experience, which persist and marginalize them until they redefine their identity. Health professionals could identify IAC in liminal states to provide appropriate support.

## Introduction

Recent decades have seen growing application of the concept of liminality to understand the complex psychological and social dimensions of serious illness. First conceptualized by Van Gennep (1969) in his anthropological theory of rites of passage, liminality describes individuals’ intermediate stages when moving between social states (e.g. being a women, and then a mother, or being a child and then an adult). Later, Turner (1969, 1979, 1994) used this construct in a socio-political analysis to describe the situation of individuals existing at society’s margins.

As it relates to transition and change in life conditions, the liminality has been seen as a relevant construct to describe transitions between health and illness in healthcare contexts. These transitions can be experienced either positively, as in recovery trajectories (Bilodeau et al., 2019), or negatively, as in the path from health to cancer diagnosis (Little et al., 1998). This construct was first used in studies concerning mental health (Barrett, 1998) or physical disabilities (Murphy et al., 1988). Then, Little et al. (1998) used this construct in psycho-oncology studies to describe and understand the experience of individuals concerned by colorectal cancer. Subsequent studies have used liminality to describe the psychological experience of individuals affected by a cancer and its treatments (IAC) at different stages of the cancer trajectories, such as during the treatment (Dawson et al., 2019), the post-treatment period (Rees, 2017), or at the end of life (Campbell et al., 2024).

In psycho-oncology, transitions related to IAC are essential to consider for clinical and research reasons. These individuals must face complex psychological, social, and physical adaptations, from initial diagnosis through treatment and beyond (Mathieu et al., 2025). Theoretical frameworks that facilitate understanding of the implications, consequences, and underlying processes during transitions are crucial. However, even though the concept of liminality seems promising in describing the transitions experienced by IAC, there is still some uncertainty about which psychological processes it encompasses. In his theory, Van Gennep (1969) has indeed only briefly defined liminality at an abstract level. The construct has thus been used quite differently from one study to another. For instance, Pietilä et al. (2018) linked liminality to biographical disruption, which is a concept that describes how chronic illness fundamentally alters one’s sense of self, social relationships, and vision of the future (Bury, 1982). Bilodeau et al. (2019) emphasized a social rupture related to the temporary loss of professional role, and Campbell et al. (2024) associated liminality with “oscillations between life and death,” which could be associated to periods where IAC hoped to be cured, and then realize that it did not happen. These examples highlights that the construct of liminality was frequently used across different contexts and associated with various concepts. Despite this relative indetermination, liminality appears increasingly common in psycho-oncology studies (Söderlund and Borg, 2018; Szakolczai, 2009); a critical examination of this construct is thus warranted to elucidate its theoretical foundations, applications, and implications for both research and clinical practice. Additionally, its conceptual heterogeneity probably impeded the development of standardized measurement tools, limiting clinical applications and research advancement. We still do not know how to measure or assess it, which hinders its clinical use and practical research applications.

Therefore, this scoping review aims to investigate how liminality is theoretically defined and applied in studies related to IAC and identify and analyze the range of concepts associated with liminality in these psycho-oncology studies, thereby mapping its conceptual landscape.

## Methods

### Protocol and registration

We followed the preferred reporting items for systematic reviews and meta-analyses extension for scoping reviews (PRISMA-ScR) checklist (Tricco et al., 2018). The protocol has been uploaded on the Open Science Framework (https://osf.io/v94yw).

### Eligibility criteria

Studies were eligible for inclusion if they were peer-reviewed and utilized or defined the construct of liminality to describe the experience of IAC. Liminality had to be explicitly mentioned in the title or abstract and applied within the article’s main text. The focus on liminality should relate to the experience resulting from a cancer experience (i.e. altered or modified self-identity, fertility preservation, return to work).

We only considered original studies written in English or French, without publication date or sample size restrictions. There were no limitations regarding the design of the studies; however we excluded meta-syntheses, literature reviews, narrative reviews, or qualitative syntheses.

The population of the empirical studies must consist of adults (18 years or older) who have experienced cancer and treatment (i.e. surgery, radiotherapy, chemotherapy, or immunotherapy). Studies focusing on children, adolescents, or the experiences of childhood cancer were excluded. This decision was made due to the potentially distinct nature of cancer experiences and consequences in pediatric populations compared to adults (Kattner et al., 2019). Regarding theoretical studies, we included those relating to cancer and liminality.

There were no distinctions concerning the cancer type or stage. Treatment may be complete or still in progress. Thus, we aimed to capture a wide range of evidence on liminality in adult cancer experiences while maintaining a focused and relevant scope for the review.

### Information sources and search strategy

A systematic literature search was conducted using the following databases: PubMed, PsycNet (including MEDLINE), Embase, Web of Science, and CINAHL (Cumulative Index to Nursing and Allied Health Literature). We also performed an additional check with the references included in the reviewed articles.

Our search strategy included index terms (e.g. MeSH terms or Emtree terms) and keywords in English and French related to liminality (equation 1) and cancer (equation 2). The final search equation combined these two equations (equation 1 AND equation 2). The research equation could be found in the Supplemental Material 1.

We limited our research to keywords appearing in the title, abstract or index terms. The retrieved references were exported as RIS (Research Information Systems) files and then imported into Rayyan software (https://www.rayyan.ai/) for further processing. No additional filters were used to limit the results of our research.

### Selection of articles and data extraction

The first and last authors independently checked each title and abstract of all retrieved articles. They verified the inclusion and exclusion criteria and the relevance of the article to the research question. After that, they compared their work and discussed their divergence until they reached a consensus. They repeated this process for the full-text screening.

All information related to the research question and pertinent data items about liminality and contextual variables were recorded in an Excel sheet. Regarding liminality, we focused on (1) the theoretical definition of liminality, (2) applications of liminality in describing IAC experience, (3) concepts associated with liminality, and (4) terms associated with liminality. Related to contextual variables, we focused on the studies’ objectives and design, cancer type, cancer stage, time since diagnosis or treatment completion, sample size, participants’ gender and age, the country where the study was conducted, and the methods used for data collection.

## Results

**Figure 1.**
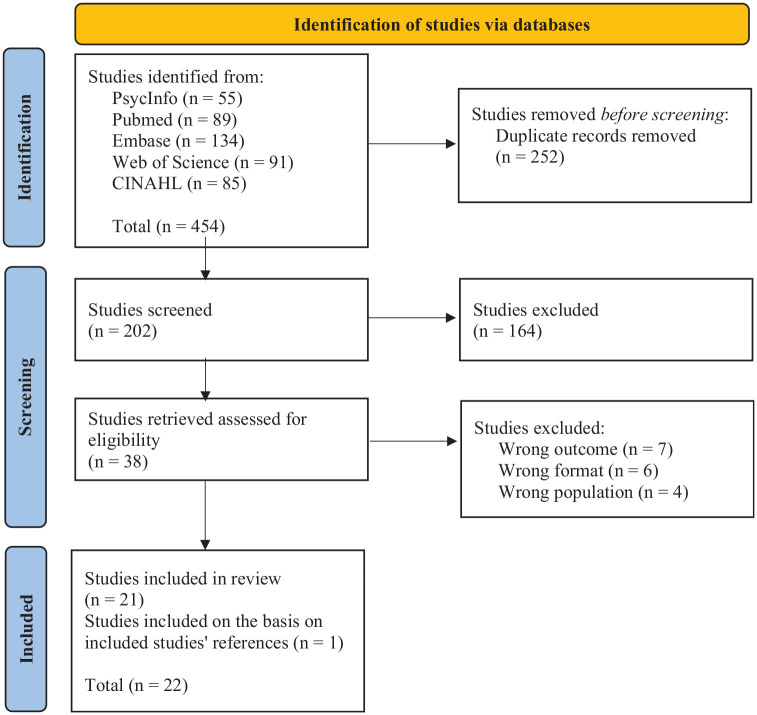
PRISMA 2020 flow diagram representing the selection resulting from the selection of studies (Page et al., 2021).

We retrieved 454 studies through the five databases. Among them, we removed 252 duplicates. We screened the title and abstract of 202 studies and excluded 164 based on our inclusion and exclusion criteria. Among the 38 studies remaining, we screened the full text and excluded seven based on a wrong outcome (i.e. the studies did not focus on liminality), six because of an incorrect format (i.e. thesis or conference abstract), and four because they did not concern only IAC and included healthy people, individual affected by other diseases, or health professionals. Based on the references of the included studies, we included one study that met our inclusion and exclusion criteria. Thus, 22 studies were finally included in order to respond to our research question. Figure 1 presents a synthesis of the different steps.

### Characteristics of the included studies

All empirical studies included employed qualitative research methods. Their publication dates span from 1998 to 2024 (mean publication year: 2015). Geographically, five studies were conducted in England, three each in Canada and Australia, two each in Greece and Belgium, and one in each Denmark, Finland, Ireland, Israel, and the United States. Sample sizes range from 8 to 878 (mean = 61, sd = 198). Across all studies, there were 910 women and 286 men participants, although one study (Dawson et al., 2019) did not report sample size or participant gender information. The ages of participants ranged from 22 to 92 years, although four studies (Cayless et al., 2010; Dawson et al., 2019; Hvidt, 2017; Koutri and Avdi, 2016) did not specify the participants’ age. Table 1 presents a summary of the empirical study characteristics. We also included two theoretical studies (Adorno, 2015; Sleight, 2016). They were published between 2015 and 2016.

**Table 1.** Characteristics of the empirical included studies.

References	Cancer type	Cancer stage	Time since diagnosis or treatment	Cancer recurrence	*n* (*n*_women_/*n*_men_)	Age in years (range, mean)	Country	Method	Aim
Avery et al. (2024)	Breast cancer (28%) Sarcoma (24%) Lung (12%) Other cancers (36%)	4	<1 to >10 years	Yes	25 (19/6)	22–39	Canada	Grounded theory	Understand how young adults navigate daily life with advanced-stage cancer and challenges to living well
Bilodeau et al. (2019)	Breast cancer	N.a.	1 months (t_0_) and more (t_1_; t_2_)	No	9 (9/0)	30–59	Canada	Interpretative phenomenological analysis	Describe the journey of individuals affected by breast cancer returning to work post-treatment
Campbell et al. (2024)	Hematological cancers	N.a.	4 months to 20 years m = 4,75 ± 6 years	Yes	21 (8/13)	51–85, *m* = 63.5	United Kingdom	Grounded theory	Understand the experience of dying of individual affected by hematology cancer
Cayless et al. (2010)	Prostate cancer	N.a.	1 year	No	10 (0/10)	N.a.	United Kingdom	Qualitative longitudinal	Explore the first year post-prostate cancer diagnosis, focusing on shifts between health and illness
Dauphin et al. (2020)	Breast cancer Gastro-intestinal cancer	N.a.	3–4 years	Yes	18 (14/4)	57–92	Belgium	N.a.	Analyze how uncertainty creates meaning for IAC
Dawson et al. (2019)	Head and neck cancers	N.a.	7–14 days post-surgery	N.a.	N.a.	N.a.	United Kingdom	Interpretative phenomenological analysis	Identify post-surgery needs of IAC and how clinical teams can meet them
Gray et al. (2005)	Prostate cancer	<4	At least 6 months of hormonotherapy	No	12 (0/12)	50–73	Canada	N.a.	Explore men’s experiences with hormone treatment
Halliday et al. (2015)	Hematological malignancies	N.a.	1–10 years	N.a.	12 (12/0)	25–39	Australia	Interpretative phenomenological analysis	Investigate fertility and motherhood experiences of young women treated for hematologic cancers
Hvidt (2017)	Breast cancer (84%) Other cancer (16%)	N.a.	N.a.	N.a.	46 (36/10)	N.a.	Denmark	Interpretative phenomenological analysis	Provide a philosophical view of the liminal transformative aspects of cancer
Jellema et al. (2021)	Breast cancer (38%) Prostate cancer (25%) Other cancers (36%)	N.a.	N.a.	N.a.	8 (4/4)	28–71, *m* = 51	Belgium	Narrative	Review the concept of liminality in cancer care, blending theory with empirical data
Koutri and Avdi (2016)	Breast cancer	1–3	<1 years	No	15 (15/0)	N.a.	Greece	Narrative	Explore how individuals affected by breast cancer create meaning through illness narratives
Little et al. (1998)	Colorectal cancer	N.a.	3–144 months median = 38 months	Yes	10 (5/5)	39–79	Australia	Grounded theory	Capture the subjective experience of illness with an emphasis on embodiment
Navon and Morag (2004)	Prostate cancer	Advanced stages	3–6 months	N.a.	15 (0/15)	57–85	Israel	Grounded theory	Understand the side effects of hormonal therapy to inform treatment policies
Parton et al. (2019)	Breast cancer (56.7%) Gynecological cancer (12.9%) Other cancers (30,4%)	Early and advanced stages	*m* = 6.22, sd = 7.01 years	N.a.	878 (693/185)	*m* = 42.5	Australia	Discourse analysis	Examine how IAC view and experience fertility preservation
Pietilä et al. (2018)	Prostate cancer	N.a.	1 year	N.a.	22 (0/22)	56–71	Finland	Narrative	Explore the experience and the means to handle the uncertainties during treatment and recovery
Rees (2017)	Breast cancer	N.a.	15 months to 9 years *m* = 3.5 years	Yes	20 (20/0)	22–44	United Kingdom	Grounded theory	Explore experiences of young women with breast cancer in the UK
Thompson (2007)	Ovarian cancer	3	2–10 years	Yes	9 (9/0)	35–59, *m* = 49.7	United States of America	Grounded theory	Convey how ovarian cancer affects IAC’s internal and interpersonal world
Trusson et al. (2016)	Breast cancer	Early stages	<12 months to >20 years	No	24 (24/0)	42–80	United Kingdom	Narrative	Analyze how women manage post-treatment challenges, including body changes related to femininity and sexuality and fear of recurrence
Wilson (2020)	Breast cancer	Early stages	T_0_: End of the chemotherapy T_1_: 6 months post-treatment	N.a.	25 (25/0)	35–48, *m* = 42	Ireland	Grounded theory	Explore young women’s experiences transitioning to survivorship after early-stage breast cancer
Ziliaskopoulou and Avdi (2023)	Breast cancer	1–3	1–9 years	No	17 (17/0)	26–57	Greece	Interpretative phenomenological analysis	Describe the mastectomy experience, including body image changes and socio-cultural impacts on identity

In terms of cancer types, six studies investigated breast cancer (Bilodeau et al., 2019; Koutri and Avdi, 2016; Rees, 2017; Trusson et al., 2016; Wilson, 2020; Ziliaskopoulou and Avdi, 2023), four focused on prostate cancer (Cayless et al., 2010; Gray et al., 2005; Navon and Morag, 2004; Pietilä et al., 2018), two on hematologic malignancies (Campbell et al., 2024; Halliday et al., 2015), and one study each addressed on colorectal (Little et al., 1998), ovarian (Thompson, 2007), and head and neck cancer (Dawson et al., 2019). Additionally, five studies covered a variety of cancer types (Avery et al., 2024; Dauphin et al., 2020; Hvidt, 2017; Jellema et al., 2021; Parton et al., 2019).

The period covered by each study varied considerably. While most studies examined the post-treatment period (*n* = 15), four studies included participants who may have been currently undergoing treatment or had recently completed it (Dawson et al., 2019; Jellema et al., 2021; Little et al., 1998; Wilson, 2020). Additionally, three studies focused on advanced cancer and the end-of-life period (Adorno, 2015; Avery et al., 2024; Campbell et al., 2024). The results below encompass findings from all studies. Where specific results pertain to a particular period (i.e. post-treatment, during treatment, or end-of-life periods), these will be explicitly highlighted.

### Results regarding liminality

Of the 22 studies reviewed, 19 referenced Van Gennep (1969) or Turner (1969, 1979, 1994) as foundational to the construct of liminality. Additionally, 14 studies cited Little et al. (1998), and six referred to Navon and Morag (2004).

Most studies described liminality as a “state” (18 studies), an “experience” (14 studies), or a “space” (8 studies), with additional terms like “stage,”“process,”“phase,” or “transition” occasionally used. Many studies applied more than one word to liminality, which underscores the conceptual indeterminacy of liminality. Furthermore, studies focusing on the post-treatment period primarily used “state” (14 out of 15) or “experience” (9 out of 15), while the three examining the end-of-life period employed “space.” Treatment period studies preferred “state” (3 out of 4) or “transition” (3 out of 4). These findings demonstrate that the conceptualization of liminality vary significantly among studies according to the cancer period. Table 2 presents these terms and their frequency by treatment periods. Further details can be found in Supplemental Material 2, which specifies each study’s preferred terminology.

**Table 2.** Terminology associated with the liminality construct and their frequencies related to the treatment period.

Words	*n* for the 22 studies	*n* post-treatment period (15 studies)	*n* during and after treatment period (4 studies)	*n* end-of-life period (3 studies)
State	18	14	3	1
Experience	15	10	2	3
Space	8	3	2	3
Stage	6	5	1	0
Process	6	3	2	1
Phase	6	3	1	2
Transition	6	2	3	1

Studies mainly employed what they considered as liminality as related to a psychological or social context, as individuals must deal with significant life changes. They highlighted liminality in what has been called “context of uncertainties and ambiguities,” relating to a variety of situations in which the person does not know what the future will be and what will follow in his/her life trajectories, or a period of vulnerabilities (21 studies), experiences related to physical, psychological, or social changes (20 studies), identity changes (17 studies), social role modifications or isolation (16 studies), and fear of death or cancer recurrence (13 studies). End-of-life studies emphasized uncertainties, death, and experience of changes. Table 3 details the contexts and concepts associated with liminality by treatment periods.

**Table 3.** Contexts and concepts associated with liminality and their frequency related to the treatment periods.

Context or concept		*n* for the 22 studies	*n* post-treatment period (15 studies)	*n* during and after treatment period (4 studies)	*n* end-of-life period (3 studies)
Contexts	Uncertainties, ambiguities, and vulnerabilities	21	15	3	3
	Experience	20	13	4	3
	Identity	17	13	3	1
	Social roles and isolation	16	11	4	1
	Fear of death or cancer recurrence	13	8	2	3
	Transition	9	5	3	1
	New normality	7	7	0	0
	Sexuality	4	4	0	0
	Fertility or motherhood	3	3	0	0
	Return to work	2	2	0	0
Concepts	Biographical disruption	10	8	2	0
	Embodiment	8	5	2	1
	Growth	7	5	1	1
	Anxiety	7	4	2	1
	Communicative alienation	4	3	1	0
	Boundedness	3	2	1	0
	Agency	2	2	0	0
	Cancer patientness	1	0	1	0

Studies often paired their hypothesized liminality construct with other concepts, relating mainly to physical or psychological challenges or positive transformations. Thus, concepts such as biographical disruption (Bury, 1982), embodiment, which relates to the importance of the body to understand, interpret and construct the lived experience (Niedenthal et al., 2005), posttraumatic growth, described by Calhoun and Tedeschi (2001) as a positive transformation that arise after a traumatic event and leads to a personal development and a better appreciation of the live, or anxiety appear to be associated with liminality. Others concepts also appeared in a few studies, such as agency, which often relates to the ability to perform actions, communicative alienation, which is described by Little et al. (1998) as the difficulties to communicate with others about its cancer experience and difficulties, boundedness, also introduced by Little et al. (1998) as the feeling to be limited by time and by its body, and cancer patientness, describing the tendency of cancer patients to adopt a social role during and after the cancer treatment (Little et al., 1998).

Most studies portrayed liminality as a negative phenomenon, where IAC faced significant challenges affecting their lives. However, eight studies (Adorno, 2015; Avery et al., 2024; Halliday et al., 2015; Hvidt, 2017; Jellema et al., 2021; Parton et al., 2019; Thompson, 2007; Trusson et al., 2016) presented a dual perspective, noting both difficulties and potential for growth, or oscillation between positive and negative experiences. One study (Dawson et al., 2019) adopted a neutral stance, describing changes without assigning a positive or negative value.

Authors highlighted characteristics of what they supposed to be liminality, particularly the uncertainty and challenges IAC face in reintegrating into their professional and social lives (Bilodeau et al., 2019; Dawson et al., 2019; Halliday et al., 2015; Jellema et al., 2021; Navon and Morag, 2004; Pietilä et al., 2018; Sleight, 2016; Thompson, 2007; Trusson et al., 2016; Wilson, 2020; Ziliaskopoulou and Avdi, 2023). Several studies also highlighted the changes in IAC’s ability to make plans (Adorno, 2015; Campbell et al., 2024; Cayless et al., 2010; Halliday et al., 2015; Hvidt, 2017; Koutri and Avdi, 2016; Little et al., 1998; Pietilä et al., 2018; Rees, 2017; Sleight, 2016), difficulties reintegrating into social roles (Adorno, 2015; Dauphin et al., 2020; Hvidt, 2017; Koutri and Avdi, 2016; Little et al., 1998; Navon and Morag, 2004; Parton et al., 2019; Ziliaskopoulou and Avdi, 2023) and challenges communicating their feelings and experiences (Halliday et al., 2015; Hvidt, 2017; Koutri and Avdi, 2016; Rees, 2017; Thompson, 2007; Ziliaskopoulou and Avdi, 2023). Some studies also found that their liminal construct could be linked with increased introspection related to IAC’s experience (Hvidt, 2017; Little et al., 1998; Thompson, 2007; Trusson et al., 2016) and an inability to accept the physical, psychological, and social changes (Gray et al., 2005; Sleight, 2016; Ziliaskopoulou and Avdi, 2023). Most of these findings emerged from studies focused on post-treatment or treatment periods, however, two studies (Adorno, 2015; Avery et al., 2024) linked the consequences of liminality to the end-of-life period.

A few studies hypothesized potential triggers for the period/state of liminality as they described it. Most of these tied to medical context, such as diagnosis (Campbell et al., 2024; Dauphin et al., 2020; Hvidt, 2017; Koutri and Avdi, 2016; Little et al., 1998), end of treatment (Bilodeau et al., 2019; Dawson et al., 2019; Rees, 2017; Wilson, 2020), the treatment side effects (Navon and Morag, 2004; Parton et al., 2019; Ziliaskopoulou and Avdi, 2023). Studies also suggested fear of recurrence (Avery et al., 2024; Halliday et al., 2015; Pietilä et al., 2018), being confronted to mortality (Adorno, 2015), or the incorporation of cancer into self-identity (Sleight, 2016) as triggers. Five studies did not specify any triggers (Cayless et al., 2010; Gray et al., 2005; Jellema et al., 2021; Thompson, 2007; Trusson et al., 2016).

Finally, regarding the duration of the period described as being liminal, ten studies suggested it is pervasive or sustained (Campbell et al., 2024; Dauphin et al., 2020; Dawson et al., 2019; Halliday et al., 2015; Koutri and Avdi, 2016; Little et al., 1998; Navon and Morag, 2004; Rees, 2017; Thompson, 2007; Trusson et al., 2016), while six (Bilodeau et al., 2019; Jellema et al., 2021; Parton et al., 2019; Pietilä et al., 2018; Sleight, 2016; Ziliaskopoulou and Avdi, 2023) indicated it could diminish over time, or being attenuated, though without specifying an exact duration.

## Discussion

The understanding of the transitions experienced by IAC and their underlying processes is central to psycho-oncology. The construct of liminality, introduced to psycho-oncology studies by Little et al. (1998), helps advance this aim. However, as our results show, liminality is applied and conceptualized differently across empirical and theoretical studies, which prevents its definition and measurement.

The concept is understood and applied differently across studies. The first example concerns the terminology associated with the construct. Nevertheless, a convergence is emerging around the words “state,”“experience,” and “space.” The term “state” highlights the transition from one condition to another caused by psychological, social, and physical changes that disturb durably the daily life, as described in several studies (Little et al., 1998; Pietilä et al., 2018; Rees, 2017). “Experience” focuses on the individuals concerned by the liminality as the construct allows to capture or describe their particular lived experience (Bilodeau et al., 2019; Hvidt, 2017; Trusson et al., 2016). “Space” situates the changes within a spatio-temporal framework (i.e. treatment and post-treatment time, or end-of-life period; Avery et al., 2024; Halliday et al., 2015; Wilson, 2020). The three components (i.e. state, experience and space) involve different conceptions of this construct. They reflect the heterogeneity of the literature. Thus, although different, these components complement each other and allow for a better understanding of the nature of the construct.

A second example of indeterminacy lies in the multiple contexts in which liminality is mentioned. The psychological domain encompasses vulnerability and fears of recurrence or mortality. The social domain includes social roles, isolation, and return to work. The physical domain covers sexuality and fertility. Finally, some studies linked liminality to abstract elements, such as transition or the emergence of a new normality. Several psychological processes associated with liminality in studies tend to confirm the contexts: psychological with anxiety, social with communicative alienation (Little et al., 1998), physical with embodiment (Niedenthal et al., 2005) or boundedness (Little et al., 1998), and identity-related with biographical disruption (Bury, 1982) or cancer patientness (Little et al., 1998). While these diverse contexts associated with liminality could be perceived as a weakness, they might also be viewed as a strength, demonstrating the construct’s transposability.

Nevertheless, there is a consensus about certain aspects of liminality, such as its period usage, triggers, consequences, and duration. Most studies covered post-treatment period. Thus, liminality appears to be mostly used to define the transition from the illness to health, even if some studies focused on the transition from illness to death. Related to the trigger, most studies agree that a major life event disrupts daily life or a biographical, psychological, social, or physical rupture exists. These ruptures may manifest as the shock of the diagnosis, overwhelming treatment consequences, or fear of death or cancer recurrence. Regarding consequences, studies have found similar results concerning the results of the transition that could lead to satisfying states, such as posttraumatic growth (Jellema et al., 2021; Trusson et al., 2016; Wilson, 2020). However, most changes are challenging, such as communication or adaptation difficulties or isolation (Little et al., 1998; Rees, 2017; Ziliaskopoulou and Avdi, 2023). Finally, regarding the duration, two-thirds of the studies considered that liminality might persist indefinitely. Liminality is not necessarily self-resolving, as suggested in several studies (Hvidt, 2017; Parton et al., 2019; Ziliaskopoulou and Avdi, 2023). These findings align with the results of Sperisen et al. (2024), indicating that IAC present several unmet needs, implying that supporting measures are necessary.

Following our analysis of the liminal construct, we can formulate a tentative definition based on the most common words, contexts, characteristics, and triggers resulting from our analysis and that concerns the transition from illness to health. Liminality could be represented as a state describing IAC’s experiences. These individuals lie in a state of vulnerability and face many uncertainties related to psychological, social, corporal, or identity changes resulting from cancer experience or fear of cancer recurrence and death. During the liminal state, they attempt to redefine their identity. A successful identity redefinition should end this state. However, the liminal state persists when the redefinition is unaccepted or incomplete. Individuals confronted with a liminal state create a new normality, isolated from the “usual” world, leading to social marginalization and struggle to make plans.

Our tentative definition aligns with Van Gennep’s (1969) description of liminality as an intermediary step between two social states, disrupting the known world. However, we oriented our definition to the possibility of liminality ending. Not all studies agreed on this point. Additionally, our tentative definition offers a framework to better understand the experience of IAC following their treatment completion. They might oscillate between feeling healed and continuing to experience illness. Nevertheless, because some IAC fare and feel better than others, it suggests that some successfully navigate their post-cancer transition (Bilodeau et al., 2019; Jellema et al., 2021). This hypothesis implies that we could support IAC, which have ended treatment, identified as being in a liminal state. Our tentative definition appears also applicable when describing end-of-life or palliative care experiences. However, as supposed by Campbell et al. (2024), the liminal state might persist until death. Our definition also encompasses IAC undergoing active treatment. Most of them experience life disruption, face numerous psychological, physical and social changes. Several are also affected by fear of death (Dawson et al., 2019; Wilson, 2020). The duration of their liminal state should extend at minimum through treatment completion. The reason is that vulnerability and uncertainty feelings should persist during the treatment. It could also be hypothesized that a temporary identity (e.g. being a patient) could be adopted. This identity subsequently requires renegotiation upon treatment conclusion, potentially initiating another liminal state.

### Limitations

When searching for the usage of liminality in cancer research, we were mainly confronted with cancer survivorship literature. International guidelines tend to describe IAC as “‘cancer survivors’ to refer to anyone who has ever been diagnosed with cancer no matter where they are in the course of their disease” (American Cancer Society, 2024). However, there is still divergence in the usage of “cancer survivors” in our field, as supported by Marzorati et al. (2017). For instance, several studies used “cancer survivors” to refer to people that end treatment. Thus, even if the liminality construct appears particularly suitable for “cancer survivors,” according to the American Cancer Society (2024), we decided not to include this keyword. It could have brought noise in our results. This is why we limited our keywords to liminality and cancer keywords. It allowed us to find studies on end-of-life or that included individuals who were in remission or still in treatment.

### Implications

Further research could apply our tentative definition in order to validate it. If such a definition is validated, it could open access to this construct’s measurement. A questionnaire measuring liminality among IAC would potentially allow the identification of those presenting difficulties in their adaptation following their cancer diagnosis or post-treatment period. Thanks to this identification, it would then be possible to support IACs in need of assistance. Research on the construct of liminality and its operationalization thus opens new ways to examine the experience of these individuals and the means used to help them.

### Conclusion

Our scoping review acknowledges the nuanced and heterogeneous applications and definitions of the liminality construct across psycho-oncology studies. This construct has been used in several periods related to IAC’s experience, such as treatment, post-treatment, or end-of-life. Despite different words, contexts, or concepts associated with liminality in psycho-oncology studies, there are some convergence points. Based on our analysis, we propose a tentative definition of liminality that captures the multidimensional experiences of IAC, highlighting the psychological, social, and identity-related challenges they face. Our tentative definition suggests that the construct of liminality does not refer to a passive state but to a dynamic process of identity reconstruction that could be strategically addressed through targeted interventions.

## Supplemental Material

sj-docx-1-hpq-10.1177_13591053251351807 – Supplemental material for Between illness and health: A scoping review of cancer experience through the construct of liminality Supplemental material, sj-docx-1-hpq-10.1177_13591053251351807 for Between illness and health: A scoping review of cancer experience through the construct of liminality by Thierry Mathieu, Nicolas Favez and Sarah Cairo Notari in Journal of Health Psychology
