# Supplementary material for: Between illness and health: A scoping review of cancer experience through the construct of liminality
Source: J Health Psychol. 2025 Jul 28;31(3):934–47. doi: 10.1177/13591053251351807 (PMC12949750; doi:10.1177/13591053251351807)
Supplement: sj-docx-1-hpq-10.1177_13591053251351807 – Supplemental material for Between illness and health: A scoping review of cancer experience through the construct of liminality [file sj-docx-1-hpq-10.1177_13591053251351807.docx]

# Supplementary materials

## Supplementary material 1

### Research equation and keywords

### Liminal keywords

Limina*

### Cancer-related keywords

Neoplasm*; Sarcom*; Cancer*; Carcinom*; Tumor; Tumour*; Néoplasme*; Sarcôme*; Tumeur*.

### Example of the research equation for Pubmed

Equation 1: Limina*[Title/Abstract]

Equation 2: Neoplasm*[Title/Abstract] OR Sarcom*[Title/Abstract] OR Cancer*[Title/Abstract] OR Carcinom*[Title/Abstract] OR Tumor*[Title/Abstract] OR Tumour*[Title/Abstract] OR Néoplasme*[Title/Abstract] OR Sarcôme*[Title/Abstract] OR Tumeur*[Title/Abstract] OR (Neoplasms[MeSH Terms])

Final equation: equation1 AND equation2, corresponding to:

(Limina*[Title/Abstract]) AND (Neoplasm*[Title/Abstract] OR Sarcom*[Title/Abstract] OR Cancer*[Title/Abstract] OR Carcinom*[Title/Abstract] OR Tumor*[Title/Abstract] OR Tumour*[Title/Abstract] OR Néoplasme*[Title/Abstract] OR Sarcôme*[Title/Abstract] OR Tumeur*[Title/Abstract] OR (Neoplasms[MeSH Terms]))

| **Supplementary material 2**  Words associated with liminality in the studies. Bold characters represent the words that are primarily used in the studies. | | | | | | | | | | |
| --- | --- | --- | --- | --- | --- | --- | --- | --- | --- | --- |
| Period | Studies | State | Space | Experience | Transition | Process | Phase | Stage | Limbo | Notion |
| After treatment period | Bilodeau et al., 2019 | **Yes** |  | **Yes** |  |  |  | Yes |  |  |
|  | Cayless et al., 2010 | Yes |  |  |  |  |  |  | Yes | **Yes** |
|  | Dauphin et al., 2020 | Yes |  | Yes | Yes | Yes | Yes | Yes |  |  |
|  | Gray et al., 2005 | **Yes** | Yes |  |  |  |  |  |  |  |
|  | Halliday et al., 2015 | **Yes** | **Yes** | Yes | Yes |  |  |  | Yes |  |
|  | Hvidt, 2017 | **Yes** |  | **Yes** |  |  |  |  |  |  |
|  | Koutri et Avdi, 2016 | **Yes** |  | Yes |  |  | Yes |  |  | **Yes** |
|  | Navon et Morag, 2004 | **Yes** |  |  |  |  |  |  |  |  |
|  | Parton et al., 2019 |  |  | Yes |  | **Yes** |  |  |  |  |
|  | Pietilä et al., 2018 | **Yes** |  |  |  |  |  | Yes |  |  |
|  | Rees, 2017 | **Yes** |  |  |  | Yes |  | Yes |  |  |
|  | Sleight, 2016 | **Yes** |  |  |  |  |  | Yes | Yes |  |
|  | Thompson, 2007 | **Yes** | **Yes** | **Yes** |  |  |  |  |  |  |
|  | Trusson et al., 2016 | **Yes** |  | **Yes** |  |  |  |  |  |  |
|  | Ziliaskopoulou et Avdi, 2023 | Yes |  | Yes |  |  | Yes |  |  |  |
| During and after treatment | Jellema et al., 2021 | Yes |  | **Yes** | Yes |  |  |  |  |  |
|  | Little et al., 1998 | **Yes** |  | Yes |  | Yes |  | Yes |  |  |
|  | Dawson et al., 2019 | Yes | Yes |  | Yes | Yes | Yes |  |  |  |
|  | Wilson, 2020 |  | **Yes** |  | Yes |  |  |  |  |  |
|  | Adorno, 2015 | Yes | Yes | Yes | Yes | Yes | Yes |  |  |  |
| End-of-life period | Avery et al., 2024 |  | **Yes** | Yes |  |  |  |  |  |  |
|  | Campbell et al., 2024 |  | Yes | Yes |  |  | **Yes** |  | Yes |  |
